# Adapting to climate change: strategies and perspectives from humanitarian health workers – A qualitative study

**DOI:** 10.1016/j.joclim.2024.100373

**Published:** 2024-12-18

**Authors:** Patricia Nayna Schwerdtle, Carol Devine, Astrid Berner-Rodoreda, Shannon A. McMahon, Kate Bärnighausen

**Affiliations:** a Heidelberg Institute of Global Health, Heidelberg University, Heidelberg, Germany; b IWR, Heidelberg University, Heidelberg, Germany; c Daldelah Institute for Global Health Research, Canada; d Médecins Sans Frontières, Canada

**Keywords:** Climate change adaptation. Climate change resilience. Climate change and health. Humanitarian assistance

## Abstract

**Introduction:**

Climate change is contributing to humanitarian health crises. However, research on the intersection of climate change and health in humanitarian settings often prioritises understanding impacts over identifying solutions. This study adopts a solutions-oriented approach, engaging humanitarians working in medical projects to explore both existing and potential adaptation strategies to mitigate the adverse health effects of climate change.

**Materials and Methods:**

The study involved 49 semi-structured qualitative interviews with humanitarian health workers from Médecins Sans Frontières (MSF) across 30 countries. Conducted in English, French, Spanish, Portuguese, and Arabic, the interviews focused on identifying adaptation solutions to address climate-related health impacts at individual, community, and organizational levels. Data were analysed using a hybrid coding approach, combining deductive and inductive techniques informed by framework analysis.

**Results and Discussion:**

The research highlights a perception of high vulnerability and low readiness to address climate change in the studied countries, exposing an adaptation gap—the disparity between adaptation needs and current efforts. Initially, participants found it challenging to identify adaptation strategies, often focussing on mitigation (emission reduction) rather than adaptation. From the adaptation activities identified, we developed an ‘Adaptation Continuum’ framework, which ranges from maladaptation to resilience-building. Additionally, we created a matrix of climate change adaptation (CCA) examples to illustrate how health risks can be addressed in contexts characterised by high vulnerability and low adaptive capacity.

**Conclusion:**

Health and humanitarian actors are witnessing the profound impacts of climate change on communities and projects worldwide. Despite ongoing efforts to adapt, there remains a lack of consensus on how to effectively operationalize these initiatives. This research introduces the ‘Adaptation Continuum’, a conceptual framework designed to guide the planning, implementation, and evaluation of adaptation activities in four key domains: knowledge and awareness, infrastructure and technological solutions, operational adaptation, and policy and advocacy.

## 1 Introduction

Climate change contributes to health and humanitarian crises, profoundly impacting population health and well-being. Rising temperatures and extreme weather events - such as heatwaves, hurricanes, and floods - contribute to increased mortality and injury rates. Additionally, climate change alters the distribution and activity of vectors, influencing the spread of infectious diseases. It also exacerbates food and water insecurity and disrupts social systems, shaping patterns of conflict and migration, all with important implications for health [1]. These interconnected effects have led to a growing number of people requiring humanitarian assistance [2], while simultaneously increasing the demand for health services and complicating their delivery. The negative impacts of climate change disproportionately affect people living in poverty, those without access to healthcare, and people living in fragile and conflict affected states.

Health systems and humanitarian agencies face mounting challenges in safeguarding communities from the direct and indirect health risks associated with climate change. Although much of the existing health research has centered on the impacts of climate change [3], less attention has been given to solutions - particularly climate change adaptation (CCA), which are efforts aimed at reducing the adverse effects of climate change - in humanitarian settings. A deeper understanding is needed of how humanitarian workers are experiencing and addressing climate change to better inform policy, shape international discourse, and drive systemic transformation [[4], [5], [6]].

The humanitarian community lack consensus on its role in addressing climate change [7]. Perspectives vary widely: some advocate for prioritizing ‘climate aid’ over traditional ‘war aid’ in response to the growing frequency and severity of climate change related disasters [8]. Others highlight the importance of prevention through greater investment in CCA and disaster risk reduction (DRR), proposing a more anticipatory role for humanitarians [5]. While some humanitarians have begun accessing climate finance to support CCA initiatives [9], others argue that building climate resilience in conflict zones requires a rethinking of humanitarian roles, not simply increasing funding [10].

These debates focus on two central concepts, which also arise in our research - climate change adaptation and resilience. Climate change adaptation is defined as "*the process of adjustment to actual or expected climate and its effects, in order to moderate harm or exploit beneficial opportunities"* [11]. Communities that invest in early warning systems, resilient infrastructure, and diversified livelihoods are better equipped to withstand and recover from extreme weather events such as floods or heatwaves [10,11]. For example, Save the Children, funded by the Green Climate Fund (GCF) and in partnership with the Lao People's Democratic Republic (PDR) government and the World Health Organisation (WHO), is enhancing climate resilience in Lao PDR's health system. This initiative supports over 1.8 million people in climate-vulnerable rural areas by enhancing early warning systems and health services [5].

Resilience refers to *“the capacity of social, economic and environmental systems to cope with a hazardous event or trend or disturbance, responding or reorganizing in ways that maintain their essential function, identity and structure while also maintaining the capacity for adaptation, learning and transformation”* [11]. Both adaptation and resilience aim to reduce vulnerability to climate change with the potential to strengthen communities, healthcare systems and humanitarian responses in climate-vulnerable settings. Resilience-building efforts, such as ecosystem restoration, social safety nets, and capacity-building programs, are needed for long-term adaptation to changing environmental conditions [12,13]. Studies highlight that proactive adaptation and resilience-building efforts reduce vulnerabilities, protect lives and livelihoods, and support sustainable development [10].

Recent climate change discussions have reignited debates about the boundaries and interplay between humanitarian assistance and long-term development [12]. If efforts to mitigate and adapt to climate change fail, the number of people requiring humanitarian aid due to climate change could double by 2050 [5], further intensifying already unmet needs [13]. To address these challenges, more resources are needed to support both emergency aid and long-term resilience building. However, substantial gaps remain in areas such as risk reduction, sustainable development, and financing mechanisms [14]. Growing consensus calls for integrating humanitarian assistance with long-term development through enhanced coordination, inclusion of local actors, improved preparedness, and greater financial flexibility [15].

Qualitative research on CCA examines how individuals, communities, and institutions respond to climate impacts [[16], [17], [18]]. This body of work highlights the social, cultural, economic, and political factors that shape adaptation strategies, highlighting both challenges and opportunities. Key themes include the role of climate risk perceptions as a pre-condition for local adaptation [19] and the exploration of issues such as heat adaptation, public health, and policy implementation [[20], [21], [22], [23]]. However, a systematic review found that CCA research in conflict-affected states often adopts a narrow focus, primarily addressing agriculture concerns in rural areas [24], while neglecting broader systemic challenges.

This study aims to address the gap in CCA research within the health and humanitarian sector, particularly in fragile and conflict-affected states where humanitarian actors are most active. By gathering insights from humanitarian health workers^1^ on observed climate impacts and potential solutions, the research seeks to identify adaptation strategies that strengthen organizational resilience and guide the development of climate-responsive policy interventions.

## 2 Materials and methods

### 2.1 Study setting

Data were collected in 30 climate-vulnerable countries in Africa, Central and Southeast Asia, Central and South America, the Middle East, Europe and the Pacific. The selected countries were identified as having high vulnerability and low readiness to address climate change as determined by GermanWatch [25] and the University of Notre Dame ‘ND-GAIN Index’ [26]. Within these countries, the interviews centre on projects delivering emergency medical humanitarian assistance.

### 2.2 Data collection

#### 2.2.1 Study design

We used an exploratory, qualitative design based on epistemic in-depth interviews. An exploratory qualitative design is suited to the research question because it can handle complex and context-specific dynamics and can capture a nuanced and comprehensive exploration of human experiences. Epistemic-type interviews emphasize the collaborative process of knowledge creation between the interviewer and interviewee and a more equal partnership, where both parties contribute to the construction of knowledge [27].

#### 2.2.2 Recruitment and sampling

We recruited participants via advertisements made through internal channels within the organisation. Participation was voluntary. We used stratified purposive sampling with two strata: geographic location and professional profile. The first strata ensured countries facing high climate-related risk were selected. The second strata of the professional profile sought a sample with a mix of medical and non-medical (support) staff. We actively prioritized locally-hired staff in the sample who were more likely to have a long memory of the people and the environment as well as socio-cultural insights. More participants volunteered than we could interview, therefore, as we sought a balance of age, sex, professional profile, and geographical origin.

#### 2.2.3 Procedures

Nine interviewers (2 men, 7 women) received training prior to data collection. This included a description of the research aims, the subject matter and the interview guide. All interviewers were university graduates in relevant fields including social sciences, medicine and public health. Most interviewers (7 out of 9) had experience conducting in-depth interviews. The interview guide was piloted and adjusted before beginning formal data collection. The average interview lasted 50 min (ranging from 40 - 70 mins). The over-arching questions directing the interview guide were:

• Impacts: How do you perceive, experience, and understand the impacts of climate change and environmental degradation in project contexts concerning health, patients, and communities?
• Adaptation: How is the humanitarian sector adapting operational responses (or not) to climate change?

Data collection took place between September 2022 and January 2023. An explanatory statement and consent form were provided in English, French, Portuguese, Spanish and Arabic. Interviews were conducted in person, via telephone, and by Zoom, depending on the participant's preference, and were audio-recorded. Participants were asked in which language they would like the interview to be conducted, and interviewers were matched accordingly. Interview recordings were transcribed verbatim using OtterAI, DeepL and HappyScribe software. Recordings were pseudonymized, cross-checked for accuracy within a week of the interview, and translated into English within four weeks. We conducted structured debriefings on almost half of the interviews. The principal investigator debriefed the initial interviews, followed by group debriefs with more than one debriefer. We began the initial analysis of the debriefing notes alongside data collection.

### 2.3 Ethical approval

Ethical approval was obtained from the Médecins Sans Frontières (MSF) Ethical Review Board (Approval no: 2234). Written informed consent was secured from all participants. Confidentiality and pseudonymity were maintained throughout data collection and analysis. Identifiable information was securely stored and accessible only to the research team.

### 2.4 Data analysis

We employed the framework analysis method for data analysis [28,29]. This structured approach involves several distinct stages. First, data familiarization was conducted through the in-depth review of transcripts and structured debriefs (one-on-one, progressive to group debriefs later in data collection) to gain a comprehensive understanding of the dataset. Following this, preliminary frameworks were identified [[30], [31], [32]] focusing on the health impacts of climate change, and climate change adaptation in the context of health. A priori themes derived from existing literature were combined with emergent themes to build analysis frameworks. The coding process involved systematically applying these frameworks to the data and assigning data segments to specific categories.

Subsequently, indexed data were charted into a matrix, enabling structured comparisons and facilitating pattern recognition. Summarizing and analysis involved scrutinizing these matrices for overarching themes and patterns. Finally, the interpretation phase allowed for the synthesis of findings into a coherent narrative, ensuring the study's objectives were met.

We employed a framework analysis approach to analyse the interview data, beginning with an immersive coding process. Rather than saturation, we used the concept of ‘information power’ [33], to decide when data collection was sufficient to answer the research question. Information power was achieved by the diversity and richness of the dataset, which included a wide range of participant experiences and perspectives from multiple disciplines and diverse medical humanitarian projects and contexts. We systematically coded the data, allowing us to identify and refine key themes organized according to the study's conceptual aims. These themes were then mapped to construct a conceptual framework (Fig. 2: The climate change adaptation continuum) based on the three features of CAA activities (timing, reactivity, and vulnerability) and four outcomes of CCA activities (maladaptive, surviving/coping, adaptation and resilience.

## 3 Results

**Fig. 1:**
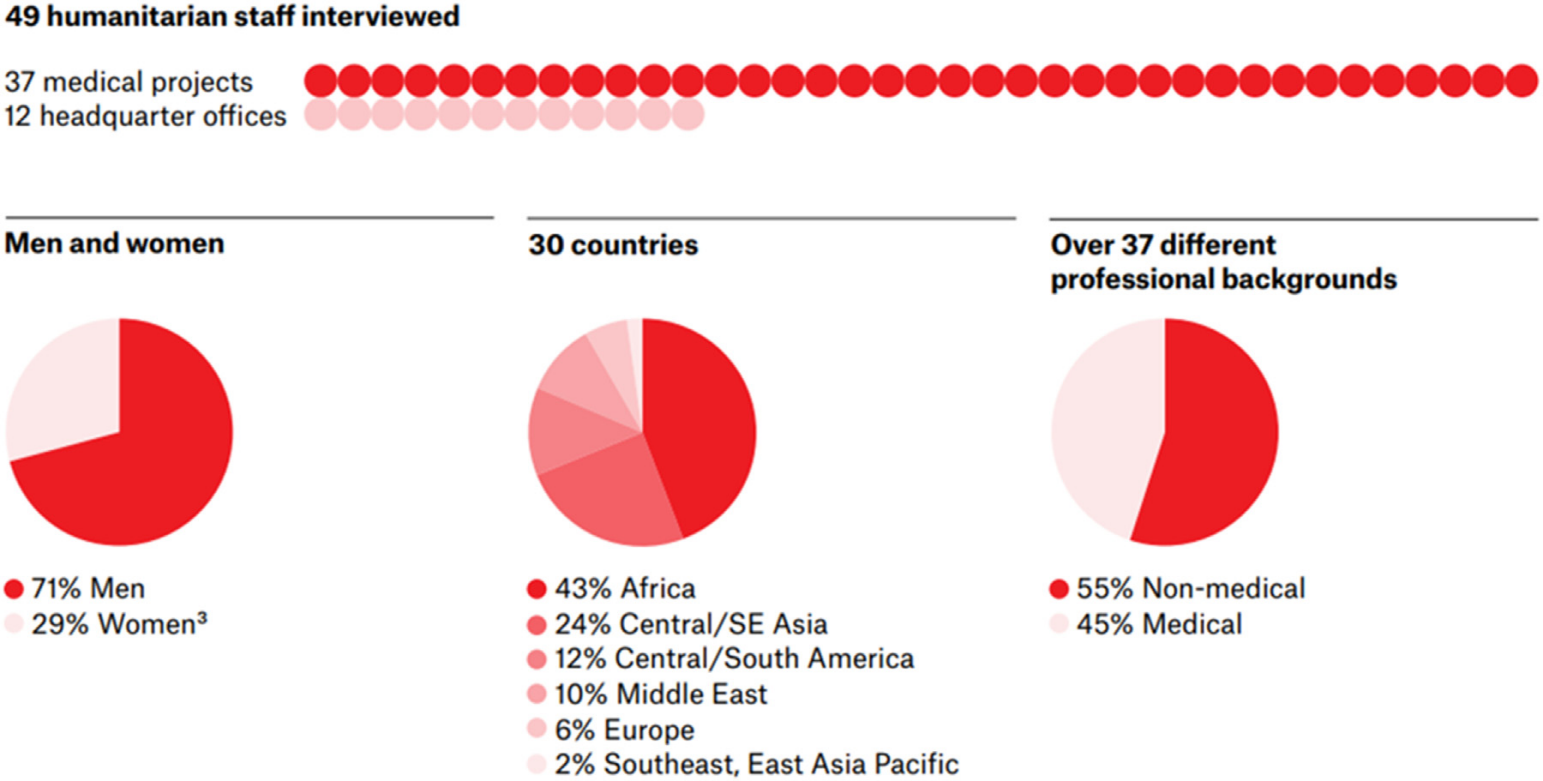
Sample description. (Credit: Figure created by The Office of Gilbert Li graphic design, 2023, Canada). Legend for Fig. 1. Demographic characteristics of the qualitative sample that comprising of 49 humanitarians (1 red dot depicts 1 interview participant), including 37 from medical emergency projects (red) and 12 from headquarters (pink). The sample consisted of 71% men (red) and 29% women (pink), with interviews conducted across 30 countries (red: Africa and different shades of red depicting the other regions). Participants represented 37 professional backgrounds, with 55% non-medical (red) and 45% medical staff (pink).

The sample was comprised of 49 staff (71 % men and 29 % women) working in 37 health projects and 12 support offices in 30 countries (43 % Africa; 24 % Central and South East Asia; 12 % Central and South America; 10 % Middle East; 6 % Europe; 2 % East Asia Pacific). The staff were 55 % medical (direct contact with patients) and 45 % non-medical staff who are also known as support staff (For example, administrative, and logistic staff) (See Fig. 1).

### 3.1 The adaptation continuum

**Fig. 2:**
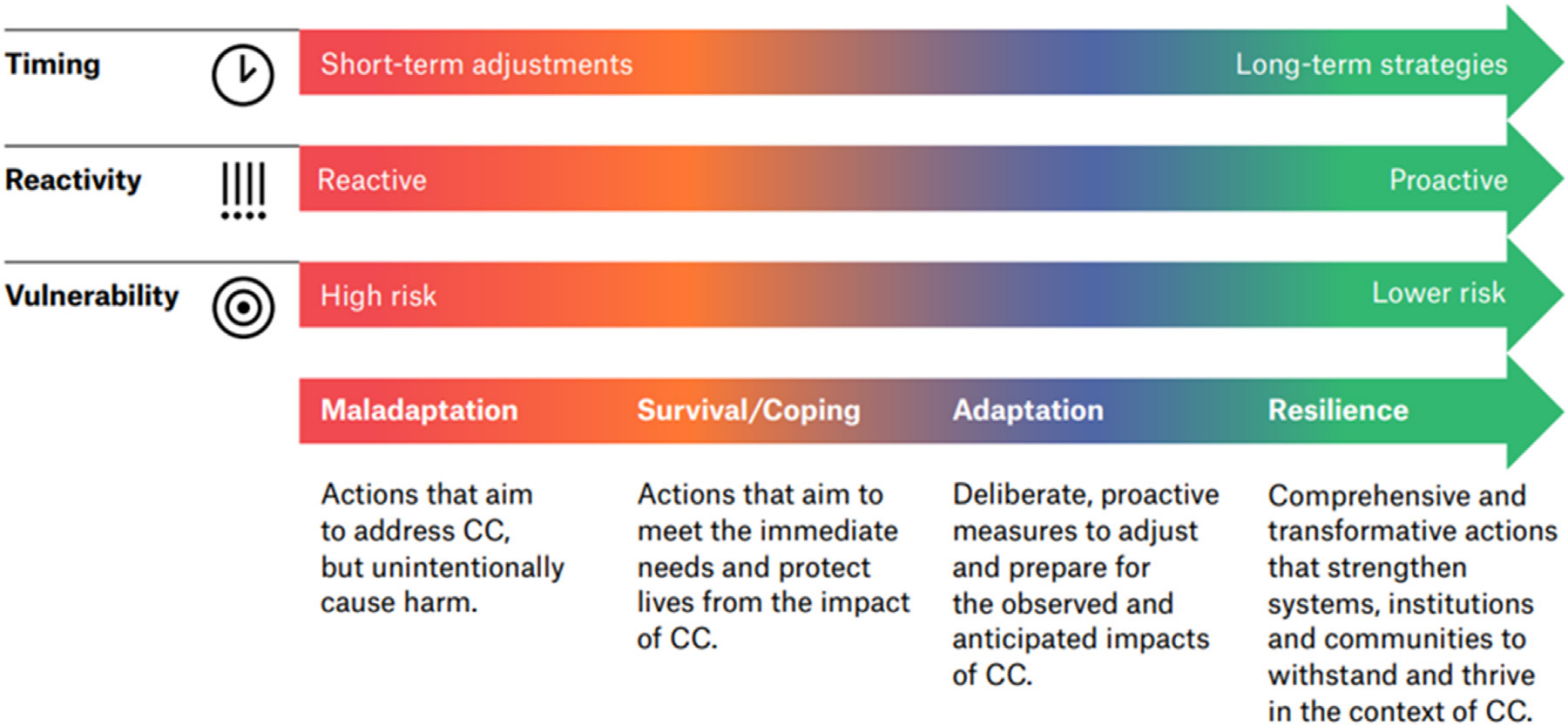
The Adaptation Continuum. (Credit: Figure created by The Office of Gilbert Li graphic design, 2023, Canada). Legend - The vertical axis highlights three key aspects of adaptation: Timing – Differentiates between short-term adjustments and long-term strategies. Reactivity – Distinguishes between reactive responses (post-climate hazards) and proactive measures (anticipating hazards). Vulnerability – Reflects the outcome of activities, ranging from increased to reduced climate-related health risks. Activities classified as maladaptive are undesirable, as they exacerbate climate-related risks. Conversely, resilience-building activities aim to reduce long-term climate risks. Together, these features capture the core dynamics of adaptation and resilience along the continuum.

We developed a conceptual framework that depicts a climate change adaptation continuum (Fig. 2). The continuum is based on the finding that CCA activities, actual and potential, as described by the participants, fall on a spectrum that spans from maladaptation to resilience-building.

### 3.2 Maladaptation

Participants described actions or strategies that, despite aiming to address climate change impacts, unintentionally exacerbate vulnerability or lead to negative consequences. This category also included inaction in the face of climate-related challenges, which participants similarly described as maladaptive.

*“Do what is practical… and also try to figure out what we are very bad at. Learn from failures, for example, in Bangladesh, a project was financed…that put boulders, big rocks, up to save the local area from being flooded…It's not only that water can't get in, but water cannot get out.”* - Advocacy Manager, Somalia, Female

Relief interventions could also lead to environmental problems as this coordinator recounted:

*“In Mozambique, people were moving from one place to another place. I had to send them a shelter, food, hygiene kit… this was causing plastic pollution, all because they were single use and nobody cares about recycling.”* -Supply Chain Coordinator, Ukraine, Female

One interview highlights an acknowledgment of climate change impacts but a lack of corresponding action, attributed to limited resources and competing priorities perceived as more urgent -

*“I mean, this conflict, this hideous war, where basic needs are not met. Believe me, nobody will relate what you were just saying about the impact of climate change… on our environment and our health… Because the reality is difficult. Basic needs are our main focus now. Even for me, as a humanitarian worker, to secure my own livelihood is an issue. To survive, and also in this difficult context, to survive from the conflict is our main concern. I don't care if the Earth is going to, you know, suffer now. I need to also avoid to suffer first, so that I can help the Earth. So, sorry for this reality but that's how it is in our country.”* - Advocacy Manager, Male, Yemen

Maladaptive practices encompass efforts to address climate change impacts that unintentionally increase risk or create new problems. Additionally, maladaptation can result from inaction driven by resource constraints and competing demands, which understandably foster a short term, emergency-focused mindset.

### 3.3 Survival and coping

These themes were interrelated, with subtle differences between survival and coping. Participants described adaptation efforts that were just above maladaptation but not yet fully adaptive. For survival, immediate actions addressed urgent climate change consequences to increase individual and community survival chances. These activities involved emergency responses like providing shelter, food, and water during extreme weather events or disasters. Participants observed community members doing all they could to survive and help others stay alive:

*“…I saw in Madagascar, there was a report where I was shown a lady who drank the urine of her cows because there was no more water…There are few options… Is it people who are poor to begin with, who live in harsh environments and then have very few coping mechanisms available to them.”* - Board Governance & Support Coordinator, Ivory Coast, Female

While the local population may alert others to climate-related hazards, the information may come too late for many:

*“Here in Sana'a, usually because it's a mountainous area, the flood comes from upstream. The people who are living upstream when this flood starts, call the people who are downstream and warn them. Usually, they do all that… But when it comes, lots of people get killed. If they didn't leave that street immediately, they will definitely drown.”* —Water and Sanitation Manager, Yemen, Female

In terms of coping, participants described how they sought to achieve more than saving lives. They broadened their aim to save livelihoods or reduce risk in some way. Coping activities were still short-term measures to deal with the immediate impacts of climate change. These activities were incremental and reactive without addressing long-term changes. Coping sometimes involved ingenuity in facing challenges such as water and food insecurity, energy impoverishment and a lack of water and sanitation facilities. For example,

*“Most of the people in very far-flung areas do not have great connectivity. Or even if they have great connectivity, it is very bad…most of the time electricity is missing. So, they go for a solar panel with a direct current fan connected to it. So, they are using those strategies, like a wet towel on their heads.”* - Logistician, Pakistan, Male

Testimonies of survival highlight the extreme and dire circumstances people face during climate change related shocks or stresses, often leaving them with few or no viable options for adaptation. As a result, many are compelled to resort to drastic measures to survive, alongside engaging in coping strategies to endure the challenges.

### 3.4 Adaptation

While survival and coping activities are crucial in immediate climate responses, participants also described more comprehensive, sustainable strategies to adapt to climate change. Some of these were aspirational, aiming to address root causes of vulnerability. For instance, linking weather data with disease surveillance enables early warning systems to provide proactive medical responses to malaria outbreaks, improving health outcomes. Participants also mentioned adaptive solutions in health-determining sectors like agriculture, such as modifying agricultural practices to withstand climate hazards and diversifying crops to supplement food supply.

*“Some households in Kenya adapt to drought and pests by growing fast maturing vegetables such as cowpeas, amaranth and managu.”* —Evaluation Manager, Kenya, Male.

Becoming self-sufficient was another strategy that local communities employed:

*“In the community . . . you see these food scarcities are there. So, more people have a small land beside their houses or a lawn. So, if they do kitchen gardening, they can provide the food for themselves.”* —Logistician, Pakistan, Male

“*Regarding malaria, as I mentioned earlier, our project in the DRC has observed, through preliminary analyses, a noticeable shift in rainfall seasonality. This change has coincided with a significant increase in malaria incidence, reaching hyper-endemic levels. As a result, for the first time in the DRC, we implemented Mass Drug Administration to address malaria.”* - Doctor, Switzerland, Male

*“So sustainable health infrastructure, medical procurement, waste management, and forecasting climate information systems around acute events. Those are some of the areas which I think need to be interconnected instead of having one siloed approach.”* —Strategic Medical Lead, India, Male

Other participants considered migration as a form of climate change adaptation:

*“The only adjustment [to adapt to climate change] I could see was the movement. There's no other thing than that, at least of the populations for which I had to set up activities. It was the departure, it was to flee as much as these populations flee violence, as much as they flee the impacts of climate change. There was no resilience mechanism that was put to help them stay.”* - Ivory Coast, Female

Adaptation comprised of actual and potential responses to climate change impacts that addressed the underlying causes of vulnerability and built mechanisms to mitigate risk in the longer term.

### 3.5 Resilience

Participants were asked how they defined resilience in the context of climate change. They described long-term activities that may not be considered within the realm of emergency responses. Resilience was largely discussed as aspirational, rather than occurring now, requiring collaboration at various levels:

*"There is a state institution .. for Disaster Management. Our organisations and others have a working group with them. We are working on the contingency plan because we already have the forecast of when we're going to have the rain. It is not something (our organisation) is going to come up with by itself in a month. It comes from collaboration with government institutions and the National Institute of Meteorology itself"* -Tuberculosis Program Supervisor, Mozambique, Male.

To achieve resilience in specific areas, adaptations need to be based on community-centred interventions:

*“In the Congo, our responses are becoming more comprehensive; a package of interventions to reduce the risk of malaria. We encourage people to use mosquito nets year-round. We involve communities in larval control. Without the community, these interventions wouldn't work — they need to identify the areas of stagnant water, reduce waste, and continue the response. You couldn't come in and do it for them. They need to participate, and it needs to be constant.”* —Health Advisor, Central African Republic, Female.

Participants expressed interest in engaging in resilience-building activities and emphasized the importance of ensuring that emergency responses do not undermine long-term resilience. Ideally, such responses could lay the groundwork for resilience-building once the acute phase of a crisis has passed. They shared examples of CCA strategies tailored to the specific hazards and impacts they observed in their unique settings. These included actions to avoid maladaptation, measures for survival and coping, and a few examples of resilience-building initiatives.

Several participants called on health and humanitarian organizations to allocate more resources and take concrete steps toward adaptation. Some noted that just as organizations have made commitments to mitigation efforts, a similar commitment and accountability should be extended to adaptation. They emphasized that adaptation should be pursued in tandem with scaling up mitigation efforts, highlighting the need for a holistic approach to addressing climate change impacts.

### 3.6 The adaptation matrix

**Fig. 3:**
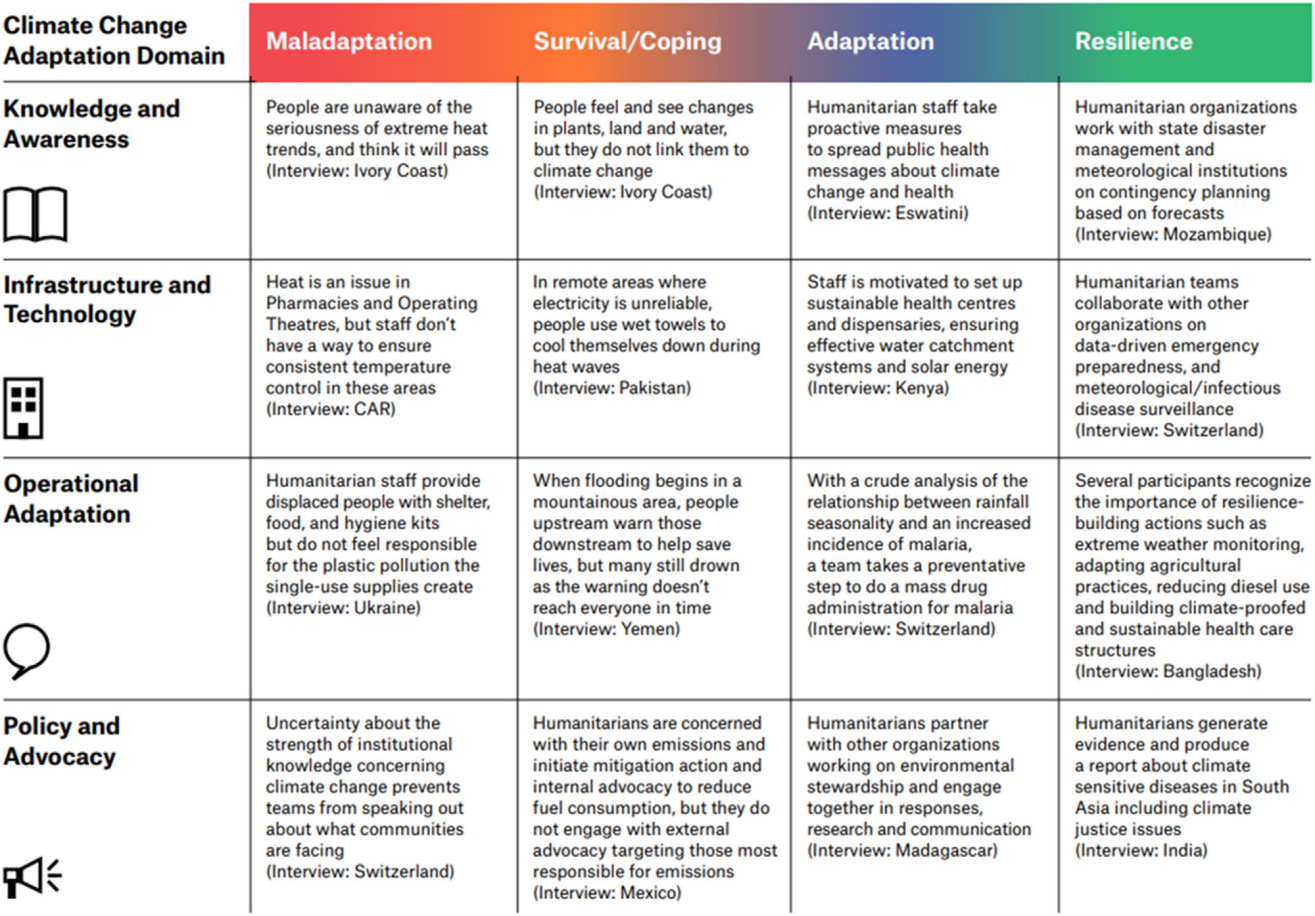
The Adaptation Matrix. Legend: This grid presents brief summaries from the interviews (not direct quotes) organized under each adaptation level on the horizontal axis, which corresponds to the four categories in Figure 2 – The Adaptation Continuum. The vertical axis outlines four domains of CCA activities, derived from the analysis of the interviews. While transcripts were not explicitly coded for actual vs. aspirational activities, participants often struggled to describe CCA action currently being implemented. As a result, the majority of the activities described were aspirational (proposed) and related to the climate-related health risks observed by participants.

The dataset also informed the development of an ‘Adaptation matrix’ (Fig. 3), providing concrete examples of CCA actions shared by participants. While some examples represent comprehensive, transformative, and potentially replicable strategies, others serve as cautionary tales, highlighting maladaptive practices.

These examples span the four levels of the adaptation continuum, from maladaptation to resilience-building, illustrating both actual and potential activities to address climate change impacts (Fig. 3).

## 4 Discussion

Our findings reveal how humanitarian health workers conceptualize climate change adaptation in contexts characterised by high vulnerability and low adaptive capacity. They highlight both actual and potential solutions to mitigate climate-related health risks in these challenging settings. Central to this research is the introduction of the ‘Adaptation Continuum’, a conceptual framework that categorizes humanitarian health activities along a spectrum from maladaptation to resilience-building. Complementing this, the ‘Adaptation Matrix’ provides specific examples of activities addressing health risks across this continuum.

The climate change adaptation continuum advances the understanding of CCA and resilience by contextualising these concepts within the humanitarian health sector. We show how a range of activities can potentially build resilience (at best) but also have the potential to increase risk – maladaptation (at worst). This framework has the potential to enrich discussions on climate change responses in the humanitarian sector and can serve as a foundation to categorise, document, evaluate and report climate change adaptation in this field.

The climate change adaptation continuum offers a starting point for addressing strategic and operational questions on how humanitarians can adapt to climate change. By defining levels of adaptation and offering examples of feasible activities, it encourages a shift in humanitarian assistance from reactive, short-term responses to proactive, resilience-building approaches. These findings align with other studies advocating for greater integration of resilience into humanitarian and health responses [4].

However, not all participants supported resilience-building within the scope of humanitarian assistance, highlighting an ongoing debate about the boundaries and interplay between humanitarian and development actions [34]. One perspective argues that humanitarian actors, whose primary mandate is to address immediate crises, often hesitate to engage in long-term development due to concerns that it could dilute their core mission of saving lives and alleviating suffering. This hesitation is further complicated by the often ambiguous transition between humanitarian aid and development, as well as persistent financial constraints.

Conversely, the Office for the Coordination of Humanitarian Affairs (OCHA) advocates for an integrated approach, suggesting that humanitarian adaptation complements development activities in the context of climate change. This approach emphasizes preparedness, anticipatory action, and faster response times [34]. It aligns with ‘The New Humanitarianism,’ which emphasizes local empowerment, resilience-building, and sustainable solutions through active community involvement and long-term impact [35], consistent with global development goals [36].

The Intergovernmental Panel on Climate Change (IPCC) Assessment Report 6 (AR6) also emphasizes the importance of the Humanitarian, Development, and Peace Nexus as a critical framework for effective adaptation, highlighting the need for coordination across sectors to address climate-related challenges comprehensively [1].

Our interpretation suggests a middle ground where humanitarian health programs address immediate needs while factoring in long-term climate impacts. This approach involves building resilience where feasible, bridging the divide between humanitarian and development efforts. Collaboration between humanitarian actors, who respond to urgent, crisis-driven needs, and development actors, who focus on long-term, sustainable improvements, is essential for ensuring that their efforts complement each another and help communities better prepare for future disasters. However, maintaining some distinction between these sectors remains important, as each brings expertise and strengths [37].

The debate on protecting the core mission of humanitarian work is closely tied to the concept of 'temporal discounting,' which explains why immediate concerns often take precedence over long-term challenges [38]. This dynamic heavily influences discussions on how humanitarian actors should address climate change, particularly in contexts marked by weak governance and economic instability. In contrast, affluent societies, with greater resources, are generally better positioned to invest in long-term planning and mitigation.

We identify three key implications for the health and humanitarian community: scaling up adaptation efforts, enhancing mitigation, and addressing the adaptation gap. First, support for adaptation in highly exposed regions should target health sectors and avoid maladaptation, ideally fostering resilience. Second, despite adaptation limits [39], increased mitigation efforts remain critical. Greater mitigation reduces the extent of climate impacts, thereby diminishing the need for extensive adaptation [40]. Third, humanitarians can help measure and report the adaptation gap by establishing mechanisms to assess unmet adaptation needs in vulnerable settings. These recommendations align with the Climate and Environmental Charter for humanitarian organizations, which includes commitments signed by several agencies [6].

This research prioritized in-depth experiences over establishing causation, which may limit the ability to isolate climate-specific impacts. Additionally, the study focused on a single organization, and the experiences of other humanitarian agencies could differ. Despite these limitations, the relatively large sample size for qualitative research enhances the robustness of the findings, making the conceptual insights potentially transferable to other humanitarian health organizations.

## 5 Conclusion

Health and humanitarian actors are witnessing the significant impacts of climate change on communities and projects worldwide. These challenges expose an 'adaptation gap', highlighting the need for more comprehensive actions to reduce risks, enhance preparedness, and advocate for protecting communities from climate change. While many humanitarian and health organizations are prioritizing climate change adaptation, there remains a lack of consensus on how to effectively operationalize these efforts.

The Adaptation Continuum offers a potential framework for planning, implementing, and evaluating adaptation activities across four domains: Knowledge and awareness; Infrastructure and technology; Operational adaptation; and Policy and advocacy. Although this continuum was developed from the study's data, its practical utility has yet to be tested and could be explored in future research.

Historically, health and humanitarian actors have demonstrated adaptability in responding to various threats, but the unprecedented scale and pace of climate change demands a more strategic, forward-looking approach. The Adaptation Continuum and Adaptation Matrix can serve as tools to help organizations transition toward more resilient and sustainable practices, supporting efforts to address the complex challenges posed by climate change.

## CRediT authorship contribution statement

**Patricia Nayna Schwerdtle:** Writing – review & editing, Writing – original draft, Visualization, Validation, Supervision, Software, Resources, Project administration, Methodology, Investigation, Funding acquisition, Formal analysis, Data curation, Conceptualization. **Carol Devine:** Writing – review & editing, Validation, Software, Resources, Project administration, Funding acquisition, Formal analysis, Data curation, Conceptualization. **Astrid Berner-Rodoreda:** Writing – review & editing, Validation, Formal analysis. **Shannon A. McMahon:** Writing – review & editing, Validation, Supervision, Formal analysis, Conceptualization. **Kate Bärnighausen:** Writing – review & editing, Validation, Supervision, Methodology, Investigation, Formal analysis, Data curation, Conceptualization.

## Declaration of competing interest

The authors declare that they have no known competing financial interests or personal relationships that could have appeared to influence the work reported in this paper. The authors declare the following financial interests/personal relationships which may be considered as potential competing interests: This work was supported by Doctors without Borders (Canada) and the Heidelberg Institute of Global Health, Heidelberg University, Germany.

## Footnotes

1 ‘We use "humanitarian" to refer broadly to individuals providing assistance in crisis-affected areas, including natural disasters, conflict, and displacement. This includes roles across the "humanitarian sector," from frontline emergency personnel to leaders influencing policy. Our focus is on "humanitarian health workers"—such as nurses, midwives, doctors, public health specialists, and logisticians—who deliver healthcare in these settings’.

## References

[1] Pörtner H.O., Roberts D.C., Poloczanska E.S., Mintenbeck K., Tignor M., Alegría A., et al. IPCC, 2022, Summary for policymakers. 2022.

[2] Baxter L., McGowan C.R., Smiley S., Palacios L., Devine C., Casademont C. (2022). The relationship between climate change, health, and the humanitarian response. The Lancet.

[3] Berrang-Ford L., Sietsma A.J., Callaghan M., Minx J.C., Scheelbeek P.F., Haddaway N.R. (2021). Systematic mapping of global research on climate and health: a machine learning review. Lancet Planet. Health.

[4] Nayna Schwerdtle P., Irvine E., Brockington S., Devine C., Guevara M., Bowen K.J (2020). Calibrating to scale: a framework for humanitarian health organizations to anticipate, prevent, prepare for and manage climate-related health risks. Global. Health.

[5] International Crescent of the Red Cross (ICRC). The cost of doing nothing [Internet]. 2019 [cited 2024 Jun 24]. Available from: https://www.ifrc.org/document/cost-doing-nothing

[6] International Crescent of the Red Cross (ICRC). The humanitarian climate and environmental charter [Internet]. 2021 [cited 2024 May 6]. Available from: https://www.icrc.org/en/document/red-cross-red-crescent-humanitarian-sector-joins-forces-tackle-existential-threat-climate

[7] Lilly D. Humanitarians still haven't agreed what they should do about climate change [Internet]. 2024 [cited 2024 Aug 21]. Available from:https://odihpn.org/publication/humanitarians-still-havent-agreed-what-they-should-do-about-climate-change/

[8] Slim H. It's time to pivot from war aid to climate aid [Internet]. The New Humanitarian; 2021 [cited 2024 Aug 21]. Available from:https://www.thenewhumanitarian.org/opinion/2021/10/25/COP26-time-to-pivot-from-war-aid-to-climate-aid

[9] Save the Children. Major climate resilience project launches in lao PDR [Internet]. 2023 [cited 2024 Jun 24]. Available from:https://www.savethechildren.org/us/about-us/media-and-news/2023-press-releases/major-climate-resilience-project-launches-lao-pdr

[10] Vazquez-Prokopec G.M., Binkley L.E., Gomez Dantes H., Berrian A.M., Soldan V.A.P., Manrique-Saide P.C. (2023). Urbanization, human societies, and pandemic preparedness and mitigation. Modern. Glob. Health Secur. Prev. Dete. Resp. [Internet].

[11] Intergovernmental Panel on Climate Change (IPCC). Glossary. In: Global Warming of 1.5 °C. An IPCC Special Report on the impacts of global warming of 1.5 °C above pre-industrial levels and related global greenhouse gas emission pathways, in the context of strengthening the global response to the threat of climate change, sustainable development, and efforts to eradicate poverty. [Internet]. [cited 2024 Nov 3]. Available from: https://www.ipcc.ch/sr15/chapter/glossary/

[12] Philips M., Derderian K. (2015). Health in the service of state-building in fragile and conflict affected contexts: an additional challenge in the medical-humanitarian environment. Confl. Health.

[13] Organisation for the coordination of humanitarian affairs (OCHA). Global Humanitarian Overview [Internet]. 2024 [cited 2024 Jun 25]. Available from: https://www.unocha.org/publications/report/world/global-humanitarian-overview-2024-enarfres

[14] Clark R., Reed J., Sunderland T. (2018). Bridging funding gaps for climate and sustainable development: pitfalls, progress and potential of private finance. Land. use policy..

[15] Eriksen S., Naess L.O., Haug R., Lenaerts L., Bhonagiri A. (2017). Courting catastrophe: humanitarian policy and practice in a changing climate. Inst. Develop. Stu. (IDS) Bull.

[16] Lusk A. Pursuing Self-determined Responses to Climate Change in the Cook Islands: exploring the Interface between government organisational directive and local community engagement with climate change adaptation. 2015

[17] Zalameda V. Climate change adaptation strategies in the philippines-a case study within the leyte region. 2015;

[18] Mallette A., Smith T.F., Elrick-Barr C., Blythe J., Plummer R. (2021). Understanding preferences for coastal climate change adaptation: a systematic literature review. Sustainability..

[19] Braunschweiger D., Ingold K. (2023). What drives local climate change adaptation? a qualitative comparative analysis. Environ. Sci. Policy..

[20] Boeckmann M. (2016). Exploring the health context: a qualitative study of local heat and climate change adaptation in Japan. Geoforum..

[21] Macassa G., Ribeiro A.I., Marttila A., Stål F., Silva J.P., Rydback M. (2022). Public health aspects of climate change adaptation in three cities: a qualitative study. Int. J. Environ. Res. Public Health.

[22] Chenani E., Yazdanpanah M., Baradaran M., Azizi-Khalkheili T., Najafabadi M.M. (2021). Barriers to climate change adaptation: qualitative evidence from southwestern Iran. J. Arid Environ..

[23] Sovacool B.K., Linnér B.O., Klein R.J. (2017). Climate change adaptation and the least developed countries fund (LDCF): qualitative insights from policy implementation in the Asia-Pacific. Clim. Change.

[24] Sitati A., Joe E., Pentz B., Grayson C., Jaime C., Gilmore E. (2021). Climate change adaptation in conflict-affected countries: a systematic assessment of evidence. Discov. Sustain..

[25] German Watch. Global climate risk index. weather-related loss events. [Internet]. 2022 [cited 2024 Nov 3]. Available from: https://www.germanwatch.org/en/cri

[26] University of Notre Dame. Notre dame global adaptation initiative (ND-GAIN). Country Index Rankings. [Internet]. [cited 2024 Nov 3]. Available from: https://gain.nd.edu/our-work/country-index/

[27] Berner-Rodoreda A., Bärnighausen T., Kennedy C., Brinkmann S., Sarker M., Wikler D. (2020). From doxastic to epistemic: a typology and critique of qualitative interview styles. Qual. inq.

[28] Furber C. (2010). Framework analysis: a method for analysing qualitative data. African J. Midwif. Women's health.

[29] Goldsmith L.J. (2021). Using framework analysis in applied qualitative research. Qual. report.

[30] Scheelbeek P.F., Dangour A.D., Jarmul S., Turner G., Sietsma A.J., Minx J.C. (2021). The effects on public health of climate change adaptation responses: a systematic review of evidence from low-and middle-income countries. Environ. Res. Lett.

[31] Schipper E.L.F. (2020). Maladaptation: when adaptation to climate change goes very wrong. One Earth..

[32] Haines A., Ebi K. (2019). The imperative for climate action to protect health. New England J. Med.

[33] Malterud K., Siersma V.D., Guassora A.D. (2016). Sample size in qualitative interview studies: guided by information power. Qual. Health Res..

[34] OCHA. Humanitarian affairs segment of the united nations economic and social council high-level panel. humanitarian action and climate change: advancing anticipatory approaches, strengthening resilience and enhancing collaboration in response to the climate crisis. [Internet]. 2021 [cited 2024 Apr 29]. Available from: https://www.unocha.org/sites/unocha/files/HLP%20on%20Climate%20Change%20-%20Concept%20Note.pdf

[35] Fox F. (2001). New humanitarianism: does it provide a moral banner for the 21st century?. Disasters..

[36] Adami M. (2021). Disorder within the humanitarian sector: the old versus new humanitarianism debate. Disasters..

[37] European Union. Linking relief, rehabilitation and development – an assessment. [Internet]. 2001 [cited 2024 Jan 29]. Available from: https://eur-lex.europa.eu/legal-content/EN/TXT/?uri=celex:52001DC0153

[38] Ruggeri K., Panin A., Vdovic M., Većkalov B., Abdul-Salaam N., Achterberg J. (2022). The globalizability of temporal discounting. Nat. Hum. Behav..

[39] Mechler R., Singh C., Ebi K., Djalante R., Thomas A., James R. (2020). Loss and Damage and limits to adaptation: recent IPCC insights and implications for climate science and policy. Sustain. Sci..

[40] Schwerdtle P.N., Devine C., Guevara M., Cornish S., Christou C., Wyns A. (2023). What cannot be mitigated or adapted to, will be suffered. Loss and damage in health and humanitarian terms. J. Clim. Chang. Health.

